# infection and vaccination of Covid-19 rates in the psychiatric department of Monastir

**DOI:** 10.1192/j.eurpsy.2023.488

**Published:** 2023-07-19

**Authors:** B. Amamou, M. Ben Mbarek, L. Gassab, I. Betbout, F. Zaafrane, L. Gaha

**Affiliations:** Psyciatry, faculty of medicine of Monastir, university of monastir, Monastir, Tunisia

## Abstract

**Introduction:**

The COVID-19 pandemic has created unprecedented challenges for the global health system. In this context, we have suggested as a research hypothesis that during this global health crisis, people with mental disorders, due to the phenomenon of “under medicalization”, would be more affected by Covid infection and would have less chance to be vaccinated.

**Objectives:**

To calculate the infection rate and vaccination rate for COVID-19 in patients with mental disorders.

**Methods:**

This is a descriptive and cross-sectional study that took place over a period of one month (from March 2, 2022 to April 2, 2022) and involved patients attending the outpatient department of psychiatry at the Fattouma Bourguiba University Hospital in Monastir.

It was conducted using a predeveloped survey with 15 questions exploring sociodemographic characteristics, history of Covid infection (personal infection, hospitalization, infection in the family, death in the family…) and vaccination for Covid-19 and its modalities.

**Results:**

The medium age of our sample was 44.9 ±13.7 years. The average duration of illness was 12.5 years and the average number of hospitalizations was 1.65.

Psychotic disorders were the most represented, 57.1%, compared to mood disorders and anxiety disorders.

Twenty-one percent (21%) of patients reported infection with COVID 19 and 3.1% required hospitalization.

Forty-six percent (46%) had an infected family member and 2.5% had a death in the family caused by COVID-19.

The rate of access to vaccination among our patients was 73.0%. The majority received 2 doses (60.0%), and they were vaccinated on their own initiative (68.0%) and by appointment (71.4%).

**Conclusions:**

The disability presented by mental disorders, particularly psychotic disorders, can expose patients to marginalization. Indeed, patients with severe mental disorders could constitute a vulnerable population to COVID-19 infection because of their difficulty in accessing care, especially during the COVID-19 pandemic, hence the recommendations.

Particular attention must always be paid to patients with mental health disorders, regarding their access to care and the promotion of health for this population.

**Disclosure of Interest:**

None Declared

